# A Voice Cloning Method Based on the Improved HiFi-GAN Model

**DOI:** 10.1155/2022/6707304

**Published:** 2022-10-11

**Authors:** Zeyu Qiu, Jun Tang, Yaxin Zhang, Jiaxin Li, Xishan Bai

**Affiliations:** ^1^Information Engineering University, Zhengzhou 450001, China; ^2^Handan Vocational College of Science and Technology, Handan 056000, China; ^3^Yunnan Minzu University, Kunming 650504, Yunnan, China

## Abstract

With the aim of adapting a source Text to Speech (TTS) model to synthesize a personal voice by using a few speech samples from the target speaker, voice cloning provides a specific TTS service. Although the Tacotron 2-based multi-speaker TTS system can implement voice cloning by introducing a d-vector into the speaker encoder, the speaker characteristics described by the d-vector cannot allow for the voice information of the entire utterance. This affects the similarity of voice cloning. As a vocoder, WaveNet sacrifices speech generation speed. To balance the relationship between model parameters, inference speed, and voice quality, a voice cloning method based on improved HiFi-GAN has been proposed in this paper. (1) To improve the feature representation ability of the speaker encoder, the x-vector is used as the embedding vector that can characterize the target speaker. (2) To improve the performance of the HiFi-GAN vocoder, the input Mel spectrum is processed by a competitive multiscale convolution strategy. (3) The one-dimensional depth-wise separable convolution is used to replace all standard one-dimensional convolutions, significantly reducing the model parameters and increasing the inference speed. The improved HiFi-GAN model remarkably reduces the number of vocoder model parameters by about 68.58% and boosts the model's inference speed. The inference speed on the GPU and CPU has increased by 11.84% and 30.99%, respectively. Voice quality has also been marginally improved as MOS increased by 0.13 and PESQ increased by 0.11. The improved HiFi-GAN model exhibits outstanding performance and remarkable compatibility in the voice cloning task. Combined with the x-vector embedding, the proposed model achieves the highest score of all the models and test sets.

## 1. Introduction

Voice cloning [1] is a speech synthesis method that allows machines to synthesize the speech of a specific target speaker. It also provides a critical technical means for generating personalized speech. In personalized human-computer interaction scenarios, voice cloning technology possesses a wide range of applications in intelligent electronic terminal equipment like autonomous robots, Internet of Vehicles, Internet of Things, etc.

This technology can be divided into two categories based on the amount of target speaker corpus used. One method is a speech synthesis method that is based on a large amount of the target speaker corpus. The fundamental principle of this method is to train a speech synthesis system with a large amount of the target speaker's speech and synthesize the voice of the target speaker. The main disadvantage is that it needs to collect a large number of speech samples of a specific person, which is a tedious job in many cases. Hence, this method is rarely used. The second approach is based on a small number of samples. There are two ways to implement this method. One method involves using the speaker adaptation method [2]. The basic idea is to obtain a more matchable acoustic model by finetuning the parameters of the trained multispeaker generation model through an adaptive algorithm. Speaker adaptation entirely depends on adaptation parameters and leads to an increase in memory storage and serving costs. The second approach is to use the speaker encoding method [3]. The basic idea is to select an independent speaker encoder to extract the embedding vector of the target speaker and then splice the speaker embedding vector into the multispeaker speech generation model for controlling the speech. Finally, the voice of the target speaker is synthesized by the vocoder. The advantage of this method is that the trained speaker encoding model does not require any finetuning, and the speaker embedding vector can be directly inferred from only a couple of speech samples of the target speaker, so the cloning speed is fast. The key part of this method is to design a good speaker encoder that has the capacity to extract the features that characterize the target speaker from a small number of speech fragments. The similarity of the cloned speech can be determined by the quality of extracted speaker features. Arik et al. made a detailed comparison between the speaker encoding and speaker adaption methods and both methods performed well in voice cloning tasks [1]. The speaker adaptation method requires thousands of finetuned steps to achieve a high-quality adaptive effect, making it more difficult for deployment in mobile devices without real-time synthesis. The cloning time and necessary memory for the speaker encoding method are really less, which is crucial for practical applications.

Although previous works in voice cloning have appropriately considered the limited speech samples in personalized voice, they have not completely addressed the key issues. They finetune the whole model [4] or the decoder part [5, 6], achieving good quality but leading to too many adaptation parameters. Reducing the number of adaptation parameters is crucial for the practical application of voice cloning tasks. Also, the memory storage can explode due to the increase in the number of users. Some works only finetune the speaker embedding or train the speaker encoder part [7, 8], which does not require any fine-tuning during voice cloning. Although these approaches lead to a lightweight and efficient adaptation, they provide poor cloning quality.

So far, Tacotron 2 [9], based on sequence-to-sequence architecture, has been a very popular model in the field of speech synthesis, possessing great development prospects and significant versatility. Tacotron 2 can be divided into two submodules: the acoustic feature prediction module and the vocoder module. At the time of training, the acoustic feature prediction module usually inputs the text sequence and outputs the acoustic features, while the vocoder module restores the predicted acoustic features to speech waveforms. However, Tacotron 2 cannot precisely control and synthesize diverse speech samples. To synthesize sounds closer to human beings, Tacotron 2 is usually extended and applied in several other tasks such as voice cloning, speech style control, speech prosody control, code-switching, etc. Wang et al. achieved prosody transfer and enhanced the emotional information of synthesized speech by adding a prosody encoder in the acoustic feature prediction module to model and learn the prosody features in both supervised as well as unsupervised ways [10, 11]. The speaker encoder can be simply regarded as a text-independent speaker recognition model. Kinnunen et al. completed speech conversion based on *i*-vector [12] in speaker recognition [13]. It is difficult to retain the nonlinear features in the original data when the *i*-vector uses a linear transformation to reduce the dimensions, which have been replaced by a *d*-vector with strong antinoise ability [14]. By adding the speaker encoder and extracting the *d*-vector with the target speaker features, the multispeaker TTS system [15] realizes the preliminary voice cloning. However, *d*-vector does not fully consider the context information of the entire utterance, which leads to the omission of speaker information. Snyder et al. first proposed a framework for extracting speaker embedding features based on the time-delay neural network (TDNN) [16] and successfully obtained the *x*-vector, which was applied to the speaker verification task and outperformed the traditional speaker vector. The application of the *x*-vector in the speaker encoder can effectively guide the prediction of acoustic features, which can significantly improve the similarity of the cloned speech. By combining WaveNet [17] based on the autoregressive model as a vocoder, Tacotron 2 can generate high-quality speech, but the sequential reasoning process of the autoregressive model makes it sluggish and inefficient to generate speech, which cannot meet the requirements of real-time applications. To address the limitations of autoregressive models, more and more researchers began focusing on nonautoregressive models based on generative adversarial networks (GAN). Parallel WaveGAN [18] and MelGAN [19] are the early attempts of GAN on vocoder. Although the model reasoning speed can be significantly improved, the speech they generated is not satisfactory in terms of quality. The appearance of HiFi-GAN [20] breaks the shackles not only by effectively modeling the long-term correlation of the speech waveform but, more importantly, by effectively modeling the periodic mode of the speech waveform. Besides, it achieves real-time and high-fidelity speech waveform generation. Moreover, as one of the most advanced vocoder networks, the HiFi-GAN model is used as the backend by many end-to-end speech synthesis systems to restore the predicted Mel spectrum to speech waveforms. However, there are still some shortcomings in HiFi-GAN, which fail to balance the speech quality with model parameters and inference speed. Therefore, the HiFi-GAN model must be improved for better application in the voice cloning task.

Although the multispeaker TTS model based on Tacotron 2 can expand the system architecture along with supporting the voice cloning function of multiple speakers, it is still slightly insufficient in terms of speaker feature extraction and synthesis speed. The speech information of the entire sentence is not taken into consideration by the d-vector, which affects the similarity of the cloned voice. The WaveNet vocoder can have a severe impact on the speed of speech generation. To better balance the relationship between speech quality, model parameters, and inference speed of the voice cloning system, the speaker features are extracted based on TDNN, and the output of each speech segment is aggregated after passing through the model through statistical pooling. This represents the feature vector of the target speaker and improves the quality of the generated speech. In this paper, a competitive multiscale convolution (CMSC) strategy and a depth-wise separable convolution (DSC) strategy are introduced to improve the HiFi-GAN model, which replaces the WaveNet vocoder, to significantly reduce the number of model parameters and further enhance the inference speed.

## 2. Methods

**Figure 1:**
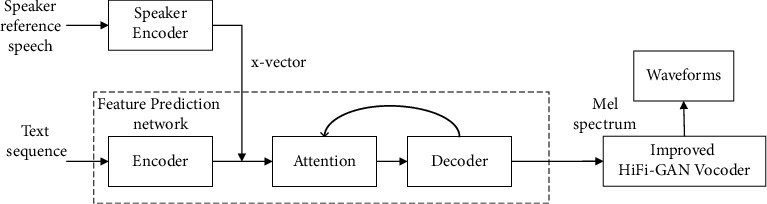
System architecture of voice cloning is based on improved HiFi-GAN.

The overall structure of the voice cloning system based on the improved HiFi-GAN model can be divided into three groups: speaker encoder network, feature prediction network, and vocoder network. As illustrated in Figure 1, a network based on speaker verification is adopted by the speaker encoder network. This encoder is trained by improving the performance of the speaker verification system. The feature prediction network follows the Tactron 2 architecture, which is implemented by an encoder-decoder architecture. First, the text sequence is converted into a semantic vector in this architecture by the encoder. After the semantic vector and the speaker embedding vector are spliced, it is processed by using the attention mechanism and forwarded to the decoder of the feature prediction network. Eventually, the decoder converts the sequence into the Mel spectrum. The vocoder network is implemented with an improved HiFi-GAN model, which quickly converts the Mel spectrum into speech waveforms while ensuring the quality of the generated speech.

### 2.1. Speaker Encoder Based on the *X*-Vector

**Figure 2:**
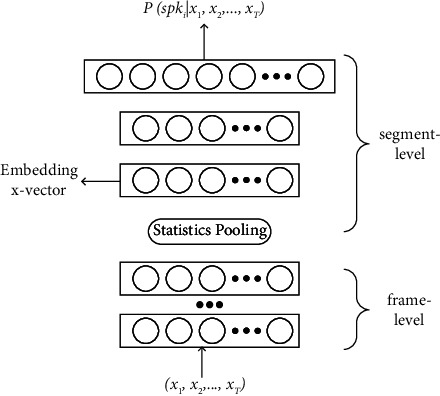
Network structure based on the *x*-vector extracted by the TDNN.

The speaker encoder network is one of the core parts of the model, which determines the similarity of the cloned speech. In this paper, the speaker verification architecture is used to implement the speaker encoder based on the *x*-vector. In the speaker verification architecture, the input is the speaker's speech feature, and the output is the speaker's discrimination information . The network structure is shown in Figure 2 [16].

The first five layers of the network are frame-level layers. The output nodes of the other layers are 512, while the output nodes of the fifth layer are 1500. The actual input is a 20-dimensional MFCC speech feature. The current frame is spliced with the input of the first and last two frames and then sent to frame 1. The corresponding total context information is 5 frames, and the input is a 100-dimensional feature vector. The three frames of {*t* − 2, *t*, *t* + 2} are spliced and sent to frame 2 as the output of the frame 1 layer. At this time, there are 9 frames including in-context information, and the splicing input is a 1536-dimensional feature vector. The operation of the frame 3 layer is similar, which comprises 15 frames' contextual information, and the splicing input is a 1536-dimensional feature vector. The output of the previous layer is directly used by frames 4 and 5 as input after batch normalization without splicing the context information. The statistical pooling layer receives the output of the frame 5 layer as input and computes the mean and standard deviation of all the frames {0, *T*} of the input speech. These two statistics are spliced together to form a 3000-dimensional vector and sent to the next two segment-level processing layers. Considering different situations, the output dimensions of segments 6 and 7 can be set, and finally, the output is sent to the output layer to obtain the probability distributions of different speakers. The specific parameters of each layer of the network are mentioned in Table 1.

A multiclass cross-entropy objective function is used to train the network to distinguish different speakers. The probability distribution of the samples corresponding to each label is obtained after normalizing the output layer by the SoftMax function. Then, the cross-entropy loss function is used to calculate the similarity of the results to the true sample probability distribution. The network parameters are continuously updated through backpropagation until convergence. As shown in (1), it is assumed that there are *K* speakers in the *N* training speech segments.*P*(spkr_*k*_|*x*_1:*T*_^(*n*)^) denotes the probability that the input speech *x*_1_^(*n*)^, *x*_2_^(*n*)^, ..., *x*_*T*_^(*n*)^ of given *T* frames corresponds to the speaker *k*. If the corresponding speaker label of speech segment *n* is *k*, then *d*_*nk*_ is 1, else, it is 0.

(1) $$\begin{array}{c} E=-\overset{N}{\underset{n=1}{\sum }}\overset{K}{\underset{k=1}{\sum }}d_{nk}\ln\left(P\left(spkr_{k}\left|x_{1:T}^{\left(n\right)}\right.\right)\right). \end{array}$$

### 2.2. Basic Structure of the Feature Prediction Network

**Figure 3:**
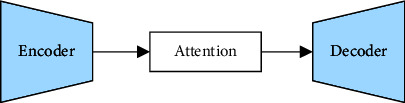
Encoder-decoder model structure.

The feature prediction network in this paper is based on the encoder-decoder model [21]. Its primary function is to direct the conversion of the input text into the Mel spectrum with the target speaker's characteristics after splicing with the *x*-vector vector output by the speaker encoder that describes the speaker's characteristics, so that the vocoder can restore waveforms.  Figure 3 depicts its fundamental architectural principle.

The encoder first models the contextual information of the input text sequence with a 3-layer convolutional network, and the output of the final convolutional layer is fed into a bidirectional long-short-term memory network with 512 units to convert the input text sequence into a high-level feature sequence. The attention mechanism computes the weight of each element in the high-level feature sequence, assigns different weights to the encoder output, performs weighted summation, and then feeds it into the decoder. In this case, the attention network employs the location-sensitive attention mechanism, which extends the additional attention mechanism [22], alleviating potential error patterns caused by the decoder repeating or ignoring some subsequences. The decoder is a 5-layer convolution postprocessing network with a 2-layer fully connected preprocessing network, a 2-layer unidirectional long short-term memory network, two linear mapping layers, and a 2-layer fully connected preprocessing network. The posterior probability of the output sequence and the output Mel spectrum are computed.

### 2.3. Improved HiFi-GAN Model

HiFi-GAN uses GAN as the basic generative model and includes a generator and two discriminators, which can efficiently convert the spectrum generated by the acoustic model into high-quality audio. HiFi-GAN is a vocoder commonly used in both academia and industry in recent years, but it still has some shortcomings. In order to reduce the model parameters of HiFi-GAN and improve the inference speed without sacrificing the speech quality, we use CMSC and DSC strategies to improve the HiFi-GAN model, and the details are described in the following submodules.

#### 2.3.1. Generator

**Figure 4:**
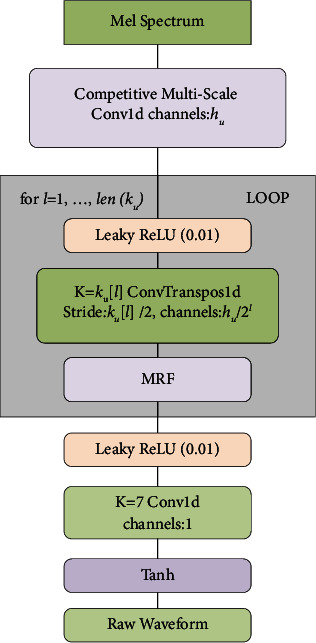
Structure diagram of the generator (*K* represents the size of the convolution kernel).

**Figure 5:**
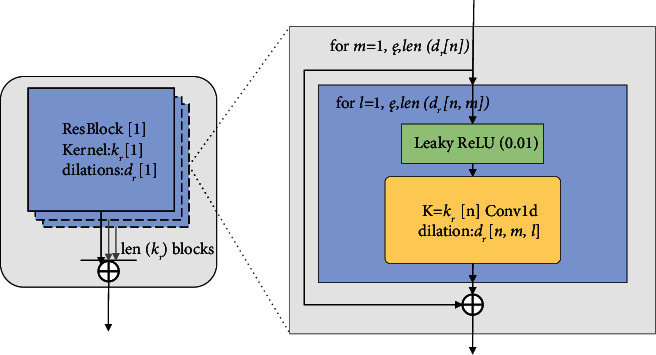
Structure diagram of MRF.

Figure 4 shows the structure of the generator, which adopts the Mel spectrum as input and continuously up-samples it by transposed convolution until the length of the output sequence is matched with the temporal resolution of the original waveform. Each transposed convolution is followed by a multireceptive field fusion (MRF) module.  Figure 5 shows the specific structure of the MRF.

The sum of the outputs of multiple residual blocks (ResBlock) is accumulated by the MRF module. Each residual block is composed of a series of one-dimensional convolutions. These convolutions have different convolution kernels and dilation rates that form different sized receptive fields, effectively modeling the long-term correlations of speech waveforms.

Unlike the original generator network, a CMSC strategy is used to extract the features from the input Mel spectrum. The multisized convolution kernels are used to process the input Mel spectrum, and the sum of these processed results is returned. Compared with the original convolutional layer with a fixed convolution kernel size to extract features from the Mel spectrum, CMSC can better capture the local features between different frames and interframe correlations of the Mel spectrum and express the feature information extracted from the Mel spectrum in a better way while providing sufficient information for the subsequent network learning. It thus improves the learning ability of the model. Besides, the original generators are composed of standard 1D convolutional layers except for a few transposed convolutional layers for upsampling. Inspired by the DSCs in images [23], in this paper, these standard 1D convolutions are replaced with 1D DSCs, which is expected to further compress the model size and speed the model inference without compromising the quality of the generated speech; making it significant for applications with limited hardware. It must be noted that DSC using weight normalization is equivalent to the depth-wise convolution and the pointwise convolution, which adopt weight normalization [24].

#### 2.3.2. Discriminator

For generative adversarial networks, the discriminator primarily plays an adversarial training role for the generator by guiding the generator to generate more realistic data. Here, the discriminator of the model basically adopts the original configuration of the HiFi-GAN model, which has two discriminators: a multiperiod discriminator and a multiscale discriminator.

#### 2.3.3. Multiperiod Discriminator

**Figure 6:**
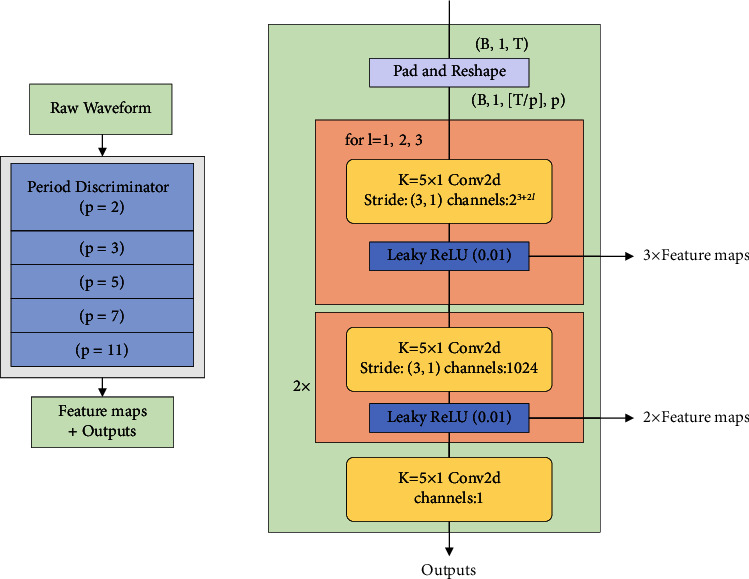
Schematic diagram of the multiperiod discriminator.

Figure 6 highlights the structure of the multiperiod discriminator (MPD). The left represents the overall structure, and the right represents the network structure of the subdiscriminator. The feature map represents the feature output of each network layer and is used in the feature matching loss in the next section. It comprises multiple subdiscriminators with the same network structure, and each subdiscriminator can capture a part of the periodic signal of the input speech to detect various potential periodic patterns in the speech data.

**Figure 7:**
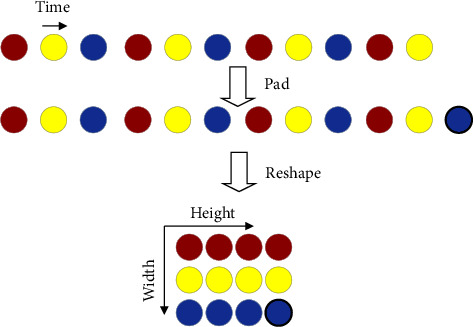
Schematic diagram of filling and reshaping in speech waveforms.

To realize that the subdiscriminator captures the periodic pattern in the speech signal, the subdiscriminators do not directly process the speech waveform but pad and reshape the speech waveform.  Figure 7 highlights the case when the period parameter *p* is 3. For ensuring that each subdiscriminator only accepts equally spaced sampling points of the input speech waveform, the interval is represented by *p*. Thus, the original one-dimensional speech of length *T* is processed into two-dimensional data with height *T*/*p* and width *p*. Therefore, the MPD needs to use a two-dimensional convolutional neural network to process these data. Other than the last network layer, the other layers use two-dimensional stride convolutions, which only stride in height, and each convolution layer uses weight normalization. In each convolutional layer of MPD, the size of the width axis of the convolution kernel is limited to 1. This leads to an independent processing of the periodic speech samples in the width axis direction. Thus, each subdiscriminator can capture the underlying periodic patterns that differ from each other in the speech by observing different parts of the speech waveforms.

#### 2.3.4. Multiscale Discriminator

**Figure 8:**
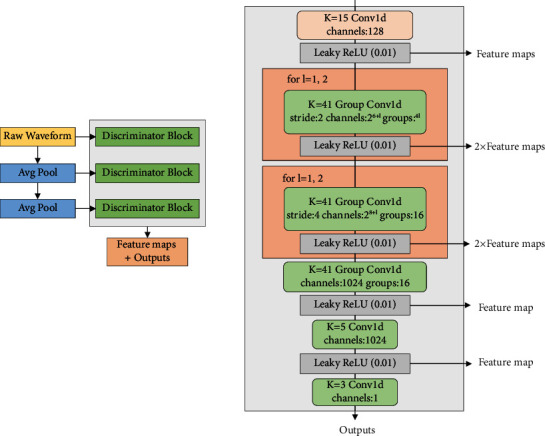
Schematic diagram of the multiscale discriminator.

Figure 8 shows the structure of the multi-scale discriminator (MSD). The left represents the overall structure, and the right represents the network structure of the subdiscriminator. The MSD is a combination of three discriminators with the same network structure but working at different scales: processing the raw speech, ×2 average-pooled audio, and ×4 average-pooled audio. To allow the use of larger-sized kernels while keeping a smaller number of parameters, the subdiscriminator employs grouped convolutions. Apart from applying spectral normalization [25] in the first subdiscriminator for raw speech processing, which is used here to help stabilize training, the other two sub-discriminators apply weight normalization.

### 2.4. Loss Function

The loss function can be categorized into three parts: adversarial loss, feature matching loss, and Mel spectrum loss.

#### 2.4.1. Adversarial Loss

For the adversarial training objectives of the generator and discriminator, the settings of LSGAN models are followed. The discriminator classifies the speech samples, where the real speech is classified as 1, and the speech generated by the generator is classified as 0. The generator generates the speech according to the input conditions to deceive the discriminator, leading to an incorrect classification of type 1 in the generated speech. Eventually, the generator can achieve the effects of mixing the spurious with the genuine through the mutual game processing between the generator and the discriminator. The adversarial loss functions of the generator and discriminator are represented in equations (2) and (3), respectively.

(2) $$\begin{array}{c} L_{A\;dv}\left(G;D\right)=E_{s}\left[{\left(D\left(G\left(s\right)\right)-1\right)}^{2}\right]\text{,} \end{array}$$

(3) $$\begin{array}{c} L_{A\;dv}\left(D;G\right)=E_{\left(x,s\right)}\left[{\left(D\left(x\right)-1\right)}^{2}+{\left(D\left(G\left(s\right)\right)\right)}^{2}\right]\text{.} \end{array}$$

In brief, MSD and MPD are described as discriminators, where *x* represents the real speech and *s* denotes the input condition (mel spectrum extracted from the corresponding real speech).

#### 2.4.2. Feature Matching Loss

To improve the ability of the generator, the feature matching loss (FML) proposed in the MelGAN model is adopted. The FML improves the generator's forgery ability by comparing the difference between the real speech and the generated speech in the output features of each layer of the discriminator network. To measure this difference, the *L*1 distance is used. The feature matching loss function formula is mentioned in equation (4).

(4) $$\begin{array}{c} L_{FM}\left(G;D\right)=E_{\left(x,s\right)}\left[\overset{T}{\underset{i=1}{\sum }}\frac{1}{N_{i}}{\left\|D^{i}\left(x\right)-D^{i}\left(G\left(s\right)\right)\right\|}_{1}\right]\text{,} \end{array}$$

where *T* represents the number of convolutional layers in the discriminator, and *D*^*i*^ and *N*_*i*_ indicate the features and number of features in the *i*-th layer of the discriminator, respectively.

#### 2.4.3. Mel Spectrum Loss

By jointly optimizing the multiresolution spectral loss and the adversarial loss, parallel WaveGAN effectively models the time-frequency distribution of real speech waveforms. Similar to the multiresolution spectral loss, the HiFi-GAN adopts the Mel spectrum loss based on the characteristics of the human auditory system, thereby improving the perceptual quality of the generated speech. Specifically, the Mel spectrum loss is the *L*1 loss in the Mel spectrum between generated speech and real waveform, as illustrated in equation (5).

(5) $$\begin{array}{c} L_{Mel}\left(G\right)=E_{\left(x,s\right)}\left[{\left\|\phi \left(x\right)-\phi \left(G\left(s\right)\right)\right\|}_{1}\right]\text{,} \end{array}$$

where *ϕ*(*·*) represents the function of extracting the Mel spectrum from speech. It must be noted that the Mel spectrum extracted here is full-band (the lowest frequency is 0 Hz and the highest frequency is half of the speech sampling rate), which differs from the band-limited Mel spectrum as the input condition. This full-band Mel loss helps the model learn the full-band frequency information of the speech.

#### 2.4.4. Total Loss

Feature matching loss and Mel spectrum loss are used as auxiliary losses to stabilize the model training and accelerate the convergence. Hence, the final loss functions used to train the generator and discriminator are mentioned in equations (6) and (7).

(6) $$\begin{array}{c} L_{G}=\overset{K}{\underset{k=1}{\sum }}\left[L_{A\;dv}\left(G;D_{k}\right)+\lambda L_{FM}\left(G;D_{k}\right)\right]+\mu L_{Mel}\left(G\right)\text{,} \end{array}$$

(7) $$\begin{array}{c} L_{D}=\overset{K}{\underset{k=1}{\sum }}L_{A\;dv}\left(D_{k};G\right)\text{,} \end{array}$$

where *D*_*k*_ represents the *k*th subdiscriminator in MPD and MSD, *λ* and *μ* denote the hyperparameters used to control the proportion of each loss, and their values are, respectively, set to 2 and 45 in this experiment.

## 3. Experimental Setup

### 3.1. Corpus

The LibriSpeech [26] speech recognition corpus is used here as the training corpus, which was widely used in the public datasets. It contains 1000 h of audiobooks at 16 kHz with corresponding texts. The audio recordings are split and sorted into shorter segments of 35 s. Also, there are two clean training sets that contain 436 h of American English speech from 1172 speakers with widely varying tonal styles. These speakers are inconsistent between the training, development, and test sets. The THCHS-30 [27] Chinese Corpus is approximately 33.5 h long, with a total of 13,388 sentences recorded by 30 college students who speak fluent Mandarin, with an average length of 20 words and an average length of 9 s per sentence. All audios in the corpus correspond to text, Chinese characters are represented by pinyin, and “spring” is represented by “chun1 tian1” (1 represents the first sound), indicating that all models are trained on prestandardized data. For accelerating the convergence of the feature prediction network, Montreal Forced Aligner is used to enforce the alignment of the audio with the corresponding text, cut silent segments longer than 0.4 s, and resegmented the data into shorter utterances.

### 3.2. Corpus Preprocessing

In LibriSpeech's training sets, one can notice ambient background noise and background noise when muted. To avoid extracting such silent segments from the complete utterance, the target spectrum is preprocessed by using spectral subtraction [28] based on voice activity detection (VAD) to identify and remove those long silences from the spectrum. For separating the silent and the nonsilent data, webrtcvad is used as the python interface for VAD, which generates a binary flag to indicate if the voice segment is pronounced. Finally, to adjust the speaker's volume, the speech waveforms are normalized.

### 3.3. Parameter Settings

Experiments are performed on a single GPU and CPU (NVIDIA Tesla V100 GPU for training, Xeon(R) E5–2620 v4 2.10 GHz CPU, and NVIDIA GTX 1080Ti GPU for testing), and the network architecture of the model is built on PyTorch.  Table 2 highlights the settings of the speaker encoder parameters based on the *x*-vector.

The key training parameters of the feature prediction network are listed in Table 3.

The model of the generator is trained with the AdamW optimizer, where *β*_1_=0.9, *β*_2_=0.999, *ε*=1*e*^−6^. After each training epoch, the learning rate decays by a factor of 0.999. The batch length of each speech is set to 16384 samples, while the batch size is tuned to 12 audio samples. The key training parameters of the generator model are listed in Table 4.

For comparative experiments, WaveNet and WaveGlow vocoders are trained with the same training and model parameters as the original settings.

### 3.4. Performance Analysis

By visualizing the speaker embedding vectors, the relationship between the speaker embedding vectors can be monitored more intuitively. The naturalness of the final cloned speech is evaluated by the mean opinion score (MOS), while the similarity is computed by the similarity mean opinion score (SMOS). In the MOS test, 20 randomly selected samples are randomly selected as an evaluation set. A group of 20 listeners who were proficient in English listened through headphones and scored according to the quality of the samples. The MOS is based on the absolute category rating scale [29], with scores ranging from 1 to 5. The MOS scores are recorded with 95% confidence intervals (CI).

## 4. Results and Discussion

### 4.1. Embedding Vector Similarity

**Figure 9:**
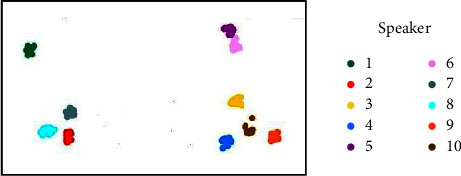
Embedding mapping based on *d*-vector.

**Figure 10:**
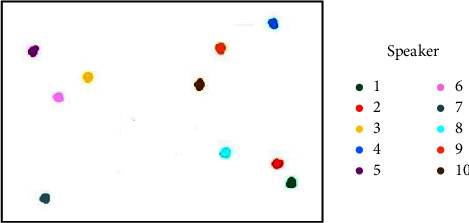
Embedding mapping based on *x*-vector.

Using the output of the trained speaker encoder, the test-other dataset in LibriSpeech is tested, which contains 10 speakers and 10 utterances from every single speaker. The speaker embeddings are visualized by reducing the dimensionality, and a clustering algorithm [30] is used to map the embeddings of 100 utterances in a two-dimensional space.  Figure 9 represents the embedding mapping of the d-vector method, and Figure 10 represents the embedding mapping of the x-vector method.

Different colors represent different speakers. It can be observed that the embedding vectors of the same speaker form the discourse clusters, and the embedding vectors of different speakers exhibit a certain distance. By comparing the two figures, it can be observed that the different speaker embeddings based on the *x*-vector are farther apart and easier to distinguish.

### 4.2. Speech Quality, Model Parameters, and Inference Speed Tests

To compare the speech quality of different vocoders, which are all trained until convergence, a perceptual evaluation of speech quality (PESQ) is used for objective evaluation, and MOS is used for subjective evaluation. Before the PESQ test, the number of sampling points is processed into an integer multiple of the frameshift by discarding the redundant silence segment at the end of each speech in the test set. Further, the real Mel spectrum from the processed test set is extracted and fed into the model to generate speech. Both the generated speech and its corresponding real speech are downsampled to 16 kHz and evaluated with PESQ using the pypesq library. Besides, the parameters of each vocoder model and the inference speed on GPU and CPU are also compared, respectively. The inference speed is measured by the reciprocal of the real time factor (RTF). RTF demonstrates the time required to generate a one-second speech. The model is considered to have a real-time capability if the time that it takes to generate a one-second waveform is less than or equal to one second. Therefore, the reciprocal value of RTF indicates that the model's inference speed is a multiple of real-time.

The results of different vocoders are listed in Table 5 for easy comparison of speech quality, inference speed, and parameters. Based on these results, it can be seen that the introduction of the improved HiFi-GAN significantly reduces the number of parameters, improves the inference speed of the model on GPU and CPU, and does not damage the quality of the speech generated by HiFi-GAN. The improved HiFi-GAN model not only reduces the number of parameters by about 68.58% but also slightly improves the voice quality (MOS increased by 0.13, PESQ increased by 0.11) when DSC and CMSC are combined. Besides, the inference speed on GPU and CPU has increased by about 11.84% and 30.99%, respectively. Compared with the flow-based WaveGlow and autoregression-based WaveNet, the improved HiFi-GAN is superior to them in all indicators.

### 4.3. Subjective Preference Evaluation

**Figure 11:**
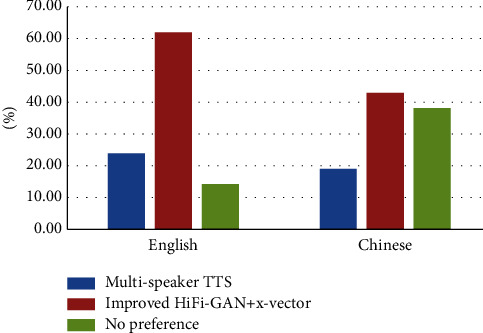
Scores of subjective preference evaluation.

Subjective preference tests are performed in this section to evaluate the model's speech cloning effect. The testers in the subjective preference comparison test are all college students majoring in the corresponding language. The testers will select their favorite sample after listening to it. They can choose to have no preference if they have no inclination.  Figure 11 depicts the test results.

The model enables voice cloning of different languages by adjusting the construction of character embedding.  Figure 11 shows that the popularity of English voice cloning samples is greater than that of Chinese voice cloning samples. The testers prefer the voice cloning system based on the improved HiFi-GAN.

### 4.4. Speech Naturalness and Similarity

To verify the effectiveness of the improved HiFi-GAN model as a vocoder on the voice cloning task, the acoustic feature prediction model is trained with the same dataset as the front end. The dataset split used to train the acoustic feature prediction model is also consistent with the dataset split used to train the vocoder network so that exaggerating experimental results because of leaking test data during evaluation can be avoided. The Mel spectrum with target speaker features is transformed into time-domain speech waveforms through the vocoder. MOS and SMOS are used to evaluate the naturalness and similarity of the cloned speech. In the test, 30 real speech samples are randomly selected and divided into three groups according to the reference corpus. One group is present in the test set from the LibriSpeech dataset, and the second group is formed in the test set from the THchs-30 dataset. The third group is present in the test set from the VCTK dataset [31] in order to verify the universality of the improved vocoder. VCTK contains 44 h of clean speech from 109 speakers, and each speaker provides more than 400 utterances in British English.

In terms of speech naturalness, the experimental results are listed in Table 6. The *x*-vector-based methods outperform the d-vector-based methods on all the models, and the improved HiFi-GAN model combined with the *x*-vector embedding achieves the highest MOS on both datasets. If data augmentation is used, the quality of the speech synthesized by the voice cloning system may be better.

In terms of speech similarity, the experimental results are mentioned in Table 7. The *x*-vector-based methods perform much better than the *d*-vector-based methods on all the models, and the SMOS is higher, especially on the LibriSpeech dataset.

It can be observed from the abovementioned two tables that the x-vector is stronger than the d-vector in representing the target speaker, and the quality of cloned speech is significantly improved. The cloned speech effect referring to the LibriSpeech corpus is better than relying on the VCTK corpus. This may be due to the fact that the training is performed in American English, and the testing is done in British English. When the experimental results in the subjective preference evaluation are combined, it is clear that the quality of English speech synthesis is superior to that of Chinese speech synthesis, which may be due to the complexity of Chinese prosodic structure and the difficulty of feature expression. The improved HiFi-GAN model combined with x-vector embedding achieves the best results in both naturalness and similarity of cloning speech, suggesting that the improved HiFi-GAN model has good compatibility in voice cloning tasks. We plan to use the Mel spectrum output of the acoustic feature prediction network under the teacher forcing condition as the input of the back-end network to fine-tune the vocoder for narrowing the difference between the real Mel spectrum and the predicted Mel spectrum.

## 5. Conclusions

In this paper, a voice cloning method with fewer parameters, faster inference speed, and higher voice quality is proposed based on the multispeaker TTS model. First, to improve the similarity of cloning speech, the x-vector feature vector that can better represent the characteristics of the target speaker is extracted based on TDNN. Then, the HiFi-GAN vocoder is improved to effectively characterize the input Mel spectrum through a competitive multiscale convolution strategy, providing sufficient feature information for the subsequent network to generate a higher-quality speech signal. Finally, the model parameters are effectively reduced by the use of depth-wise separable convolution, and the inference speed is improved without degrading the quality of the generated speech. According to the experimental results, the method in this paper effectively reduces the parameters of the HiFi-GAN model and improves the generated speech quality (MOS increased by 0.13, PESQ increased by 0.11), and the model inference speed on GPU and CPU is increased by about 11.84% and 30.99%, respectively. This proves to be very meaningful for deploying the model to application scenarios with insufficient hardware conditions and limited memory and for improving the adaptability of the model. The improved HiFi-GAN model has remarkable performance and good compatibility on the voice cloning task and achieves the highest combined score combined with x-vector embedding in all the tests.

## Figures and Tables

**Table 1:** Detailed parameters of TDNN.

Layer	Layer context	Total context	Input × output
Frame 1	[*t* − 2, *t* + 2]	5	100 × 512
Frame 2	{*t* − 2, *t*, *t* + 2}	9	1536 × 512
Frame 3	{*t* − 3, *t*, *t* + 3}	15	1536 × 512
Frame 4	{*t*}	15	512 × 512
Frame 5	{*t*}	15	512 × 1500
Sats pooling	[0, *T*)	*T*	1500*T* × 3000
Segment 1	{0}	*T*	3000 × 512
Segment 2	{0}	*T*	512 × 512
SoftMax	{0}	*T*	512 × *K*

**Table 2:** Speaker encoder model parameters based on *x*-vector.

Initial learning rate	0.0001
Model embedding size	256
Model hidden layer size	256
Model layers	3
Speaker batch size	32
Number of utterances per speaker	10

**Table 3:** The key training parameters of the feature prediction network.

Dimensions of the speaker embedding vector	256
Silence duration (s)	0.4
Utterance duration (s)	16
Mel spectrum channel number	80
Initial learning rate	0.003
Final learning rate	0.00005
Spectral window length (ms)	50
Spectral window shift (ms)	12.5

**Table 4:** The main training parameters of the generator.

Initial learning rate	0.0002
*h*_*u*_	512
*k*_*u*_	[16, 16, 4, 4]
*k*_*r*_	[3, 7, 11]
*d*_*r*_	[[[1, 1], [3, 1], [5, 1]], [[1, 1], [3, 1], [5, 1]], [[1, 1], [3, 1], [5, 1]]]

**Table 5:** Parameters, inference speed, MOS, and PESQ scores of different vocoders.

Vocoder	MOS (CI)	PESQ	Parameters (M)	Speed on GPU	Speed on CPU
Ground truth	4.56 ± 0.08	4.48	—	—	—
WaveNet	3.97 ± 0.06	3.35	—	×0.002	—
WaveGlow	3.96 ± 0.07	3.19	—	×5.26	×0.13
HiFi-GAN	4.25 ± 0.07	3.63	13.94	×70.34	×2.42
Improved HiFi-GAN	4.38 ± 0.06	3.74	4.38	×78.67	×3.17

**Table 6:** MOS of cloning speech naturalness of different models.

Metric	Settings	LibriSpeech	VCTK	THchs-30
MOS (CI)	Multispeaker TTS	3.93 ± 0.06	3.57 ± 0.07	3.64 ± 0.05
	Multispeaker TTS + *x*-vector	4.02 ± 0.08	3.72 ± 0.09	3.78 ± 0.07
	WaveGlow + *d*-vector	3.85 ± 0.06	3.49 ± 0.08	3.47 ± 0.06
	WaveGlow + *x*-vector	3.93 ± 0.07	3.74 ± 0.08	3.69 ± 0.08
	HiFi-GAN + *d*-vector	4.21 ± 0.10	3.86 ± 0.06	3.92 ± 0.07
	HiFi-GAN + *x-*vector	4.30 ± 0.07	4.15 ± 0.07	4.13 ± 0.09
	Improved HiFi-GAN + *d*-vector	4.28 ± 0.09	4.06 ± 0.05	4.11 ± 0.04
	Improved HiFi-GAN + *x*-vector	4.36 ± 0.06	4.28 ± 0.08	4.28 ± 0.06

**Table 7:** SMOS of cloning speech similarity of different models.

Metric	Settings	LibriSpeech	VCTK	THchs-30
SMOS (CI)	Multispeaker TTS	3.56 ± 0.07	3.18 ± 0.06	3.25 ± 0.08
	Multispeaker TTS + *x*-vector	3.91 ± 0.06	3.44 ± 0.07	3.59 ± 0.06
	WaveGlow + *d*-vector	3.55 ± 0.09	3.11± 0.09	3.32 ± 0.07
	WaveGlow + *x*-vector	3.89 ± 0.08	3.47 ± 0.09	3.64 ± 0.05
	HiFi-GAN + *d*-vector	3.82 ± 0.05	3.38 ± 0.07	3.43 ± 0.09
	HiFi-GAN + *x*-vector	4.15 ± 0.07	3.61 ± 0.08	3.68 ± 0.08
	Improved HiFi-GAN + *d*-vector	3.99 ± 0.10	3.52 ± 0.06	3.61 ± 0.05
	Improved HiFi-GAN + *x*-vector	4.23 ± 0.06	3.80 ± 0.08	3.84 ± 0.07

## Data Availability

The data that support the findings of this study are available from from LibriSpeech corpus, THchs-30 corpus, and VCTK corpus, which are publicly available.
